# Pharmacological Effects of Grifolin: Focusing on Anticancer Mechanisms

**DOI:** 10.3390/molecules27010284

**Published:** 2022-01-03

**Authors:** Abdelhakim Bouyahya, Aicha El Allam, Ikrame Zeouk, Douae Taha, Gokhan Zengin, Bey Hing Goh, Michelina Catauro, Domenico Montesano, Nasreddine El Omari

**Affiliations:** 1 Laboratory of Human Pathologies Biology, Department of Biology, Faculty of Sciences, Mohammed V University, Rabat 10106, Morocco; boyahyaa-90@hotmail.fr (A.B.); elallamaicha@gmail.com (A.E.A.); 2 Pharmaceutical Industry Laboratory, National Agency of Medicinal and Aromatic Plants, Taounate 34025, Morocco; ikramezeouk20@gmail.com; 3 Laboratoire de Spectroscopie, Modélisation Moléculaire, Matériaux, Nanomatériaux, Eau et Environnement, CERNE2D, Faculté des Sciences, Mohammed V University, Rabat 10106, Morocco; douae.taha02@gmail.com; 4 Physiology and Biochemistry Research Laboratory, Department of Biology, Science Faculty, Selcuk University, 42130 Konya, Turkey; gokhanzengin@selcuk.edu.tr; 5 Biofunctional Molecule Exploratory (BMEX) Research Group, School of Pharmacy, Monash University Malaysia, Bandar Sunway, Subang Jaya 47500, Malaysia; goh.bey.hing@monash.edu; 6 College of Pharmaceutical Sciences, Zhejiang University, Hangzhou 310058, China; 7 Department of Engineering, University of Campania “Luigi Vanvitelli”, Via Roma 29, 81031 Aversa, Italy; 8 Department of Pharmacy, University of Naples Federico II, via D. Montesano 49, 80131 Naples, Italy; 9 Laboratory of Histology, Embryology and Cytogenetic, Faculty of Medicine and Pharmacy, Mohammed V University, Rabat 10100, Morocco; nasrelomari@gmail.com

**Keywords:** grifolin, pharmacodynamic, anticancer effect, NF-κB, apoptosis

## Abstract

Grifolin is a volatile compound contained in essential oils of several medicinal plants. Several studies show that this substance has been the subject of numerous pharmacological investigations, which have yielded interesting results. Grifolin demonstrated beneficial effects for health via its multiple pharmacological activities. It has anti-microbial properties against bacteria, fungi, and parasites. In addition, grifolin exhibited remarkable anti-cancer effects on different human cancer cells. The anticancer action of this molecule is related to its ability to act at cellular and molecular levels on different checkpoints controlling the signaling pathways of human cancer cell lines. Grifolin can induce apoptosis, cell cycle arrest, autophagy, and senescence in these cells. Despite its major pharmacological properties, grifolin has only been investigated in vitro and in vivo. Therefore, further investigations concerning pharmacodynamic and pharmacokinetic tests are required for any possible pharmaceutical application of this substance. Moreover, toxicological tests and other investigations involving humans as a study model are required to validate the safety and clinical applications of grifolin.

## 1. Introduction

Medicinal plants’ secondary metabolites have demonstrated major pharmacological properties such as antibacterial, anti-parasitic, anti-fungal, anti-diabetic, anti-cancer, anti-inflammatory, and neuroprotective effects [1,2]. These effects are due to the presence of bioactive compounds such as terpenoids, flavonoids and phenolic acids [2,3,4,5,6]. Plant extracts can act in a pleiotropic way on several cellular and molecular targets, which justifies their diversity of action on the different systems that induce pathologies that affect humans [7,8,9].

**Figure 1:**
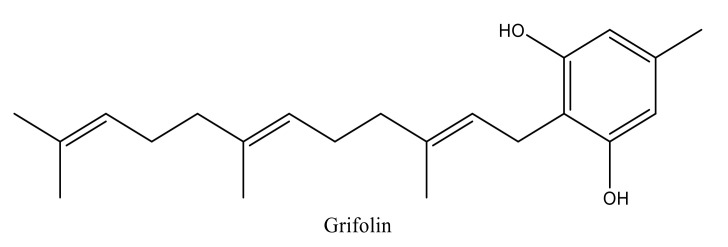
Chemical structure of grifolin designed by Chem-Draw.

Terpenoids are volatile compounds secreted by aromatic plants as secondary metabolites. These compounds exhibit many biological and pharmacological properties [10,11,12,13]. Among these substances, Grifolin (5-methyl-2-[(2E,6E)-3,7,11-trimethyldodeca-2,6,10-trienyl] benzene-1,3-dio) is a terpenoid compound with an alcoholic aromatic core (Figure 1). It is a natural bioactive compound found in medicinal plants and mushrooms and represents a major candidate for the development of pharmaceutical drugs. The natural resources that are responsible for the synthesis of grifolin are numerous and include *Peperomia galioides, Rhododendron dauricum, Solanum lycopersicum,* and *Albatrellus ovinus* [7,8,9].

Recent pharmacological investigations on natural grifolin (isolated from medicinal plants and mushrooms) and on syntheted/hemi-syntheted grifolin have shown interesting results in different biological investigations, particularly anti-bacterial, anti-fungal, anti-parasitic, as well as anti-cancer, and other effects [14,15,16,17,18].

Recently, different in vivo and in vitro investigations have shown that grifolin exhibits remarkable anticancer effects due to its capacity to react on well-defined cellular and molecular targets controlling cell transformation. Moreover, by its action on subcellular, cellular and molecular levels, grifolin can induce different signaling pathways involving several checkpoints. Similarly, it can block other signaling pathways inducing and promoting cell proliferation, transformation and invasion. A terpenoid can have the same pleiotropic effects on already cancerous cell lines and can activate essential molecular targets to induce cell transformation, promotion, and angiogenesis.

Grifolin has recently been shown to have the capacity to bind to different targets of cancer cells such as apoptosis inducing factors, CDK cyclin kinase controlling cell cycle arrest and proliferation, factors controlling DNA repair during cell division, induction of autophagy and inhibition of angiogenesis inducing systems through these different molecular mechanisms. These different checkpoints are mediated often by the activation of a second messenger transcriptional factor such as NF-κB, which is widely implicated in cancer via its ability to promote cell proliferation, differentiation and surviving.

By its cellular and molecular effects, grifolin can prevent tumor transformation at an early stage. It can inhibit or block the promotion process of cancer cells, and it can inhibit the appearance of metastasis via its important anti-angiogenic action. All these molecular mechanisms show that grifolin could be a real candidate as a drug used in anti-cancer chemotherapy but also as a molecule that could prevent the appearance of cancer effects [14,15,16]. Moreover, grifolin showed also an antibacterial, antifungal and anti-parasitic effects and can therefore be developed as antibiotic against microbial infections [17,18].

According to recent investigations, it has also been demonstrated that grifolin limits microbial growth of different human pathogenic strains, including bacteria, fungal and some *Leishmania* species.

The purpose of this review is to discuss and explore the pharmacological properties of grifolin against various internal and external human pathologies by specifying the molecular mechanisms involved, in addition to future suggestions and strategies for therapeutic interactions to enhance its use at a clinical level.

## 2. Research Methodology

Data of all studies on grifolin were collected, organized, discussed and highlighted in this review. The collection of data was carried out using scientific databases, including Google Scholar, PubMed, SpringerLink, Web of Science, Scopus, Wiley Online, ScienceDirect, and Scifnder. Data were organized according to each biological activity and then discussed and highlighted.

## 3. Results and Discussion

### 3.1. Sources of Grifolin

Grifolin is the major compound in extracts of several aromatic plants and mushrooms (Table 1).

Grifolin is the major compound in stem and leaves extract of *Peperomia galioides* of the region Callejon de Huaylas [7] and the village of Unduavi, Yungas. Other works mentioned the richness of this species in grifolin [19], in extract of aerial parts (leaves, flowers, stem) of *Peperomia galioides* [8], *Solanum lycopersicum L.* of Mexican regions characterized by the richness in grifolin [23]. In addition, grifolin is the major compound of the extract leaves of *Rhododendron dauricum* [20], leaves and twigs in Da-Hi-Shan County of Liaoning Province in China [9], and leaves of *Rhododendron dauricum* in Central Siberia and the Baikal region [22].

Moreover, methanolic extract of leaves the endemic plant *Kayea assamica* has shown that grifolin is the major compound of this plant collected from Podumoni, Lakhimpur district of Assam, India during spring period [24].

Grifolin is an isolate of the fungus *Albatrellus confluens* [29]. It is the major compound of extracts of mushrooms in *Albatrellus dispansus* [18], *Albatrellus caeruleoporus* [28], *Albatrellus flettii* [27] and *Albatrellus ovinus* [26].

Compound richness is influenced by several factors, including geographic origin, plant parts, and stages of development. Researchers have demonstrated this variability between regions, and they suggest that plants respond to the environment by fluctuating their phytochemical levels

### 3.2. Pharmacological Properties

**Figure 2:**
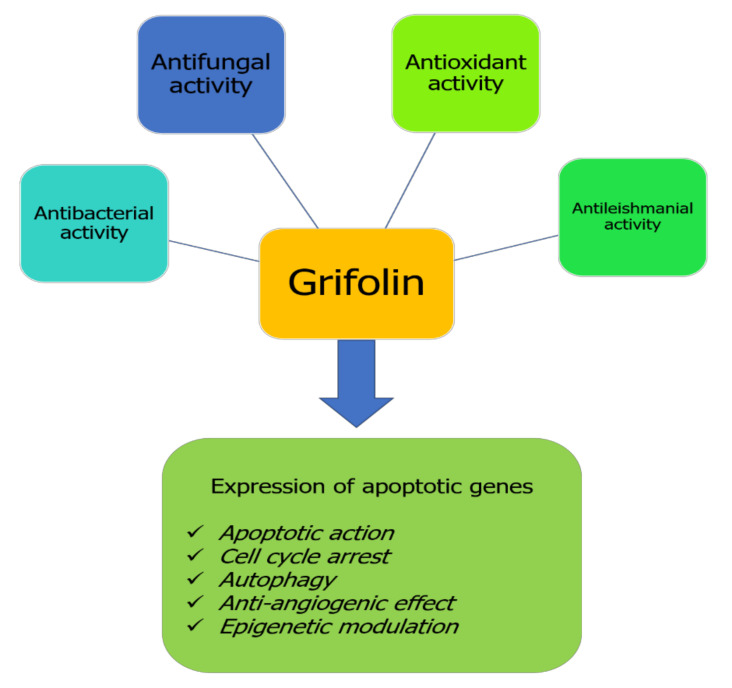
Biological properties of grifolin.

Despite having been identified and isolated from time immemorial, pharmacological studies investigating the effects of grifolin remain new compared with other natural bioactive compounds. Generally, only some pharmacological properties presented in Figure 2 have been explored for the health benefits of grifolin. This terpenoid compound exhibits particularly anticancer effects by its capacity to target different checkpoints inducing cell transformation and promotion (Figure 2).

#### Anticancer Activity

Grifolin anticancer activity has aroused the interest of several researchers who have evaluated its direct impact on different targets and signaling pathways involved in oncogenesis (Table 2) [14,15,16,30,31,32,33,34,35,36,37,38,39,40,41].

**Figure 3:**
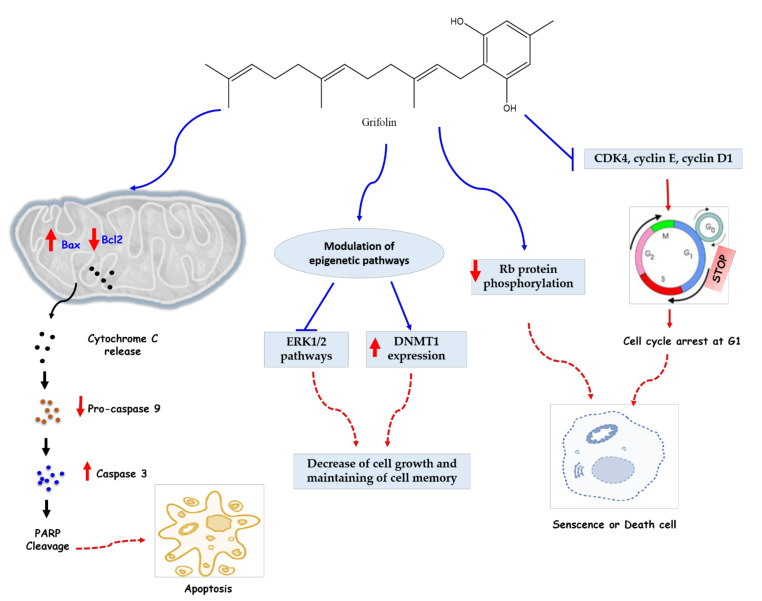
Anticancer mechanisms of grifolin. Grifolin can mediate its anticancer actions through three fundamental mechanisms. (1) Grifolin can induce an increase of Bax and a decrease of Bcl2 in Mitochondria which exhibit a release of cytochrome C and an eventual increase of caspase 3 cleave PAPR proteins, thus inducing intrinsic apoptotic action. (2) Grifolin can inhibit the ERK1/2 pathway and increase the expression of DNMT1, which plays a key role in maintaining of cell memory with inhibiting cell proliferation. (3) Grifolin can induce senescence of cancerous cells through cell cycle arrest (via inhibiting inhibition CDK4, cyclin E, and cyclin D1 regulator proteins) and a decrease of RB phosphorylation. Abbreviations: DNMT1, DNA Methyltransferase 1; ERK1/2, extracellular signal-regulated kinases 1 and 2; Bcl, B cell lymphoma; Bax, BCL2-associated X protein; RB, retinoblastoma protein; PARP, poly (ADP-ribose) polymerase; CDK, cyclin-dependent kinase.

The anticancer investigation of grifolin had started in 2005 by Ye et al. [30], who evaluated the anti-apoptotic action against human cancer cell lines. This molecule considerably inhibited the growth of several tumor cell lines, namely B95-8, Raji, K562, SW480, MCF7, HeLa, and CNE1 with IC_50_ values of 24, 27, 18, 27, 30, 34, and 24 μmol, respectively, and induced apoptosis of four cells (SW480, MCF7, HeLa, and CNE1), which was demonstrated by the analysis of the flow cytometry and morphology of the apoptotic cells by fluorescent staining. After 12 h of treatment, the mitochondria of the human nasopharyngeal carcinoma cell line CNE1 released cytochrome c (cyto c), an enzyme associated with cellular respiration and apoptosis. In addition, the activity of caspase-8, 9, 3 was increased, which means that caspase represents a key mediator of the observed apoptotic pathway. The *Bcl-2* family has also been involved in this pathway through the upregulation of Bax (pro-apoptotic protein) and the decrease in the expression of *Bcl-2*, a pro- or anti-apoptotic protein, which led to an increase in the *Bax/Bcl-2* ratio, characteristic of sensitive cells (Figure 3).

The anticancer actions of grifolin against cancer cell lines was determined by other works, such as the study of Ye et al. [34], who evaluated the effects of this terpenoid on genes expression of the main factors involving in cancer checkpoints. In this study, it was revealed that the anti-tumor effect of this molecule on CNE1 cells is explained by the inhibition of the MAP kinase pathway, ERK1/2, as well as by cell-cycle arrest in G_1_ phase and the increase in *p19^INK4D^* (a tumor suppressor gene). The mechanisms responsible for these results were inhibition of CDK4, cyclin E, cyclin D1, and decreased Rb protein phosphorylation. Cyclin D1 is one of the fundamental regulators of the cell cycle and exerts its activity in association with CDK4 by Rbp phosphorylation. CDK4 is considered to be a therapeutic target for several adenocarcinomas. The effect observed on CNE1 cells occurred mainly via the ERK1/2 pathway (Figure 3), which is involved in cell differentiation [42] and activation of the extrinsic/intrinsic apoptotic pathway [43].

This is in agreement with the results of Luo and collaborators, who recorded an inhibition of ERK1/2 kinase activities in human cancer cell lines (HeLa and MCF7) and in a cell-free system by directly binding to ERK1/2, confirmed by molecular modeling of ERK1/2 [36]. In addition, evaluation of the transcription factor Elk-1 may be an effective means in exploring the mechanism of action of this monoterpenic compound on the ERK1/2 pathway. A decrease in Elk-1 phosphorylation with a downregulation in the level of DNMT1 mRNA has been observed in some metastatic cancer cells, namely MDA-MB-231 and 5-8F, treated with grifolin. Binding to the *DNMT1* promoter region and the transcriptional effect of Elk-1 were also inhibited. This suggests that the anticancer activity of grifolin is mediated by epigenetic reactivation of genes associated with the inhibition of ERK1/2-Elk1-DNMT1 signaling (Figure 3). This secondary metabolite further exhibited anti-metastatic activity by suppressing the adhesion, motility, and invasion of metastatic cancer cells. This activity was determined by a decrease in filopodia structures that are responsible for cell adhesion and motility [44] in other cancer cells (MGC803, MDA-MB-231, and 5-8F) treated with this metabolite (40 μmol) for 24 h. At this dose, grifolin also exerted an anti-invasive activity on these cells. To investigate in vivo the anti-metastatic activity of this terpenoid, a test was performed in mice made metastatic by intravenous injection of 5-8F-Z cells. Therefore, daily treatment of 32 mg/kg of grifolin for 25 days significantly reduced lung metastases to 18.2%.

Likewise, the anticancer potential of this molecule has been tested in the management of human ovarian cancer (OC) by other research teams, [37,41]. Che et al. [37] not only evaluated the role of grifolin in the treatment of this type of cancer, but they demonstrated, for the first time, the involvement of autophagy in human OC (SKOV3 and A2780) cells treated with grifolin [37]. For this objective, cell proliferation and the autophagic effect were evaluated, as well as the main proteins of the Akt/mTOR/S6K pathway relating to autophagy. Effectively, in several tumor cells, inhibition of this pathway is linked to autophagy [45,46]. The results showed that treatment with grifolin had several positive effects on both human OC cells, such as inhibiting cell proliferation, inducing autophagic cell death, and decreasing levels of Akt, mTOR, p70S6K, S6, and 4E-BP1 phosphorylated form, suggesting that the autophagic cell death observed in this study is certainly due to the inhibition of Akt/mTOR/S6K pathway. One year later, grifolin decreased the expression levels of ERK1/2 and Akt in human OC cell lines (A2780) [41]. Moreover, for periods of 24, 48, and 72 h, grifolin (0, 25, 50, 75, and 100 µM) significantly reduced the viability of the cells studied in a time- and dose-dependent manner with a blockage of their cycle in G_1_ phase. During 24 h of treatment at the different concentrations, this molecule induced apoptosis of A2780 cells in a concentration-dependent manner. In addition, the expression of cell cycle proteins (CDK4 and cyclin D1) and proteins linked to apoptosis (cleaved-caspase-3 and cleaved-PARP, Bax, Bcl-2) were affected by this treatment. In addition to this ability to inhibit ERK1/2 kinase activities, Luo and colleagues also showed in another study [38] that grifolin may stop tumor progression by targeting other signaling pathways. This substance has shown promising anti-tumor effects, such as inhibition of cell adhesion and migration, a decrease in ATP levels, and reduction in ROS production in metastatic cells (5-8F and MGC-803 cells).

Cancer cells need high levels of ATP for their life cycle, containing high amounts of ROS responsible for the majority of their invasive effects [47]. In response to metabolic stresses, PGC1α (a transcriptional co-activator) is able to bind to specific transcription factors to regulate their function. In this case, Fra-1 and LSF, transcription factors associated with metastases, as well as the protein level of PGC1α, were attenuated following grifolin treatment. In addition, the activity of matrix metalloproteinases (MMP-2) and adhesion molecules CD44, which are highly expressed in tumor cells, was blocked by this treatment. All these results indicate that the anti-tumor properties of grifolin are linked to the inhibitory power of the interplay between Fra-1/LSF-MMP2/CD44 and PGC1α signaling.

It is remarkable that Luo, with his various co-authors, are the researchers who have best defined the underlying mechanisms of action of this secondary metabolite [14,36,39]. In 2011, grifolin increased the activity and expression of DAPK1 (tumor suppressor enzyme) in CNE1 cells via p53. This increase can induce cellular changes associated with death [48,49]. Activation of p53 by various stimuli increases the expression of DAPK1 gene. These effects have also been noted in tumor cells (MCF7 and SW480) derived from human colon cancer and human breast cancer. The apoptotic effect of this natural farnesyl phenolic compound is therefore attributed to its ability to upregulate DAPK1 via p53. Another study was focused on the epigenetic effect of grifolin in Epstein-Barr virus (EBV)-positive nasopharyngeal carcinoma (NPC) by targeting DNMT1 [14]. The pathogenesis of NPCs is associated with the latent membrane protein 1 (LMP1), encoded by EBV, responsible for several signaling pathways that induce metabolic reprogramming, epigenetic modification, invasiveness, immune escape, transformation, and cell proliferation [50,51,52]. The results of this experiment showed that treatment with grifolin attenuated glycolytic flux by inhibiting the activity and expression of *DNMT1* with its mitochondrial retention in cells.

In the same year, a derivative of this anti-tumor compound, grifolic acid, was also tested on rat pituitary adenoma cells, GH3 cells, in order to assess its activity towards the involvement of the G protein-coupled receptor 120 (GPR120), a long-chain fatty acid receptor [15]. For this reason, the authors measured the death of GH3 cells and their MPP, as well as the intracellular NAD/NADH ratio and cellular ATP levels with monitoring of GPR120 expression in these cells. After 1 h of incubation, grifolic acid, at a dose of 20 μmol/L, impaired cell viability, and 6 h later, it caused total cell death. This inhibition was dose- and time-dependent. At 24 h of treatment, it displayed an IC_50_ of 4.25 μmol/L. The involvement of GPR120 in this cell death was noted by its expression in GH3 cells. However, after 24 h of incubation, agonists of this receptor (TUG891, GW9508, and EPA) did not affect cell viability. Over time, grifolic acid significantly reduced the production of ATP levels and MMP in GH3 cells at the dose of 10 and 20 μmol/L, respectively. This treatment significantly increased the intracellular NAD/NADH ratio; this showed that the reduction in MMP was due to the decrease in NADH production. From all these findings, it can be deduced that the cell death of GH3 adenoma induced by grifolic acid was related to the inhibition of ATP production explained by the inhibition of NADH production through a mechanism independent of GPR120.

Furthermore, the mechanism of action by which this natural substance acts as an anti-tumor agent has also been elucidated by Jin et al. [32] in human osteosarcoma MG63 and U2OS cells. The authors observed induction of apoptosis, inhibition of proliferation, release of cytochrome c, decrease in mitochondrial membrane potential, activation of caspases 3/9 and cleavage of PARP in cancer cells treated with grifolin (50 μmol). These results were explained by the ability of grifolin to suppress the GSK3 and FOXO transcription factor and Akt phosphorylation.

In another context, in addition to grifolin (4) and neogrifolin (5) (mother compounds), Song and his colleagues were able to purify three new derivatives of these substances (1–3) from a methanolic extract of wild mushroom *Boletus pseudocalopus* in order to evaluate their cytotoxic effects against three cancer cell lines, namely human lung cancer A549 cells, human melanoma SK-Mel-2, and mouse melanoma B16F1 cell lines [33]. Using the colorimetric sulforhodamine B method, all compounds exhibited significant anticancer activities against mouse melanoma B16F1 and human lung carcinoma A549 with IC_50_ values ranging from 3.5 to 7.3 μg/mL and 5.0 to 10.5 μg/mL, respectively, compared to the positive control (cisplatin) with IC_50_s of 9.5 and 5.2 μg/mL. These cytotoxic effects were corroborated by the results of the antioxidant activity revealed by the DPPH test.

In contrast, this natural biological product has shown interesting antiproliferative effects, In vitro, against gastric cancer [39,40]. Using human gastric cancer cells (SGC-7901 and BGC-823), Yang and co-workers marked an inhibition of the MAP kinase pathway (MEKK3, MEK1, MEK5), explained by the decrease in expression levels of genes related to this pathway in cells treated with grifolin (50 μmol) [39]. By targeting a famous cell cycle regulator, cyclin-dependent kinase inhibitor 2D (cdkn2d), grifolin could regulate the cell cycle of gastric cancer cells. Additionally, analysis of ERK1/2 and ERK5 phosphorylation status, activated respectively by MEK1 and MEK5. Ye et al. [34] showed an inhibition of their activity and consequently an inhibition of tumor invasion [53], further demonstrated in this study. Besides, decreased cell viability and blockage of cell cycle progression in G_1_ have significantly shown that grifolin treatment induces suppression of cell proliferation with induction of cell cycle arrest in G_1_ phase. In addition, treatment with 10 and 50 µM grifolin induced apoptosis of SGC-7901 and BGC-823 cells. This was confirmed by induction of cytochrome c passage from mitochondria to the cytosol accompanied by increased activation of caspases-3 and 9.

One year later, using the same cell lines (SGC-7901 and BGC-823), these results were fully supported by a study conducted by Wu et al. [41], such as decreased expression of MEKK3, MEK1, and MEK5, upregulation of cdkn2d, suppression of cell invasion, induction of G_1_ cell-cycle arrest, and activation of caspases-3 and 9 [40]. In parallel, these gastric cancer cells were xenografted on nude BALB/c mice (16–20 mg) in order to study in vivo the anticancer potency of grifolin. Consequently, administration of a dose of 15 mg/kg b.w of this substance, every 2 days for one month, significantly increased the survival rate of all tumor xenograft animals.

The ability to inhibit cell viability by grifolin indicated in previous studies recently interested a Canadian research team [16]. After having determined a strong anti-cell viability effect of the ethanolic extracts from terrestrial polypore *Albatrellus flettii*, grifolin and neogrifolin were identified and isolated using several techniques (nuclear magnetic resonance, mass spectrometry and bioassay-guided fractionation). Grifolin exhibited remarkable cytotoxic activity against two human colon cancer cell lines HT-29 and SW-480, and one human cervical cancer HeLa cell line with IC_50_ values of 35.4 ± 2.4, 27.4 ± 2.2, and 30.7 ± 1.0 μmol, respectively. While neogrifolin displayed IC_50_ values of 34.6 ± 5.9, 24.3 ± 2.5, and 30.1 ± 4.0 μmol against HT29, SW480, and HeLa cells, respectively. This indicates that these two compounds have an anti-cell viability effect against both types of cancer cells. Furthermore, inhibition of oncogenes could be another effective treatment method by directly inhibiting their inducing effect of cell proliferation via inhibition of their biosynthesis, expression, and function [54,55]. This alternative treatment was of interest to the authors of this study [16], by targeting the expression of KRAS (an onco-target) in HT-29 and SW-480 cells. In SW-480 cells carrying the KRAS mutation, grifolin and neogrifolin inhibited KRAS expression after 48 h of treatment at a dose of 50 μmol. Regarding HT-29 cells carrying the wild-type KRAS, both compounds suppressed *KRAS* expression, similarly, at doses of 20 and 50 μmol.

Conversely, the relationship between cancer and reactive nitrogen species has been investigated in several research studies [56,57,58,59]. Nitrogen monoxide or nitric oxide (NO) can induce both angiogenesis and genotoxicity. Excessive NO production can involve mutant p53 cells, upregulate VEGF (induction of tumor angiogenesis), and upregulate p53, PARP, and DNA-PK leading to modulation of tumor DNA repair mechanisms. In this context, the inhibitory power of NO production of certain grifolin derivatives isolated and identified from *Albatrellus caeruleoporus* methanolic extract was evaluated in vitro in RAW 264.7 cells [31]. Therefore, neogrifolin, grifolin, and grifolinones A and B showed inhibitory activity with IC_50_ values of 23.3, 29.0, 23.4, and 22.9 μmol, respectively.

Taken together, grifolin and its natural derivatives may be promising anti-tumor agents. However, further studies are clearly needed to decipher the exact mechanism(s) by which these compounds act.

### 3.3. Antibacterial Activity

Grifonin’s efficacy has been reported in several studies in the pharmacological literature. It has been widely recognized, since antiquity, for its promising antibacterial proprieties.

Hirata and Nakanishi [60] have shown that isolated grifolin from *Grifota confluent* proved to be active against both *Staphylococcus aureus* and *Bacillus subtilis* without lethal effects on mice.

The susceptibility of acid-fast bacteria such as Mycobacterium avium and *Mycobacterium phlei* to this substance was also particularly noteworthy, which could be explained by the chemical structure of grifolin, which consists, among other things, of two hydroxyl groups, two p-nitrobenzoyl groups, and an aldehyde group. Nevertheless, the Gram-negative bacteria such as *Bacillus anlhracis*, *Bacillus dysenteriae*, and *Salmonella typhimurium* were resistant.Grifolin was isolated from *Albatrellus dispansus* and tested against a list of Gram-positive and Gram-negative bacteria emphasizing a more interesting effect than the reference antibiotic, the gentamicin [17]. The chemical synthesis of this antibiotic using orcinol and farnesol was successful and identical to the natural product [61], allowing its possible development into a pharmaceutical.

### 3.4. Antifungal Potency

In addition to the inhibitory effect of bacteria, Hashimito et al. [17] also reported the antifungal capacity of grifolin against *Aspergillus niger* and *Candida albicans* with a higher inhibitory power than the reference antifungal, nystatin. In the same vein, Luo et al. [18] confirmed the antifungal activity of grifolin not only in vitro but also in vivo, showing that grifolin displayed interesting inhibitory effects to mycelial growth of *S. sclerotiorum*, *F. graminearum* in addition to the spore germination of *P. oryzae* in vitro. Moreover, it exhibited a high level of curative activity against plant disease of *E. graminis* in vivo.

### 3.5. Leishmanicidal Potential

Fornet et al. [19] have evaluated the leishmanicidal activity of grifolin isolated from *Peperomia galioides*, a plant belonging to piperaceae family, against *L. donovani*, *L. braziliensis* in vitro, and against *L. amazonensis* in vitro and in vivo. Grifolin exhibited leishmanicidal properties in vitro against all three strains tested. However, the treatment of BALB/c mice infested with *L. amazonensis* has not shown any evolution in comparison to the untreated group.

### 3.6. Other Biological Properties

In addition to its pharmacological properties, grifolin isolated from different species exhibits other important biological activities. Obtained from *Albatrellus ovinus*, grifolin inhibited the TRPV1 receptor involved in the sensing of changes in the proximity of cells. In vitro, the IC_50_ values of TRPV1 receptor inhibition were law expressed in µM range with Hill coefficients between 1.5 and 2.4. In addition, grifolin was able to demonstrate in vivo effects by reducing skin reddening and microcirculation, as well as stinging and burning sensations [25].

Grifolin, which was produced via bioguided fractionation from Rhododendron dauricum extract, dramatically reduced histamine release from rat peritoneal mast cells stimulated by compound 48/80. The structure activity relationship (SAR) was deeply studied highlighting that the presence of an orcinol-connected isoprenyl side chain was important to inhibit the histamine activity [20]. In the same terms of SAR, Sugiama et al. [62] have evaluated the hypocholesterolemic action of grifolin regarding its structure on rats fed. They have shown that farnesyl orcinol was mandatory for the hypocholesterolemic action, and grifolin effect could be elicited, at least in part, via the augmented excretion of cholesterol into the feces and largely dependent on the presence of exogenous cholesterol in the diet.

In another study, the effect of grifolin against brain injury in an acute cerebral ischemia rat model was investigated. Results have shown that grifolin treatment prevented damage acting on the oxidative stress parameters by enhancing SOD and GPX, reducing MDA and NO levels in tissue homogenates of the cerebral ischemic rats, decreasing the altered levels of inflammatory mediators namely cytokines and NF-κB, in addition to reducing caspase 3 and ATP levels in the tissue homogenate of cerebral ischemic rats [32].

## 4. Conclusions

This work highlighted the sources and biological properties of grifolin. This natural bioactive compound exhibits in vitro and in vivo activities on different microbes such as bacteria, fungi and parasites. It has shown important results, while its mechanisms of action have not been well elucidated on these microbes. Therefore, further pharmacodynamic investigations on grifolin alone and/or combined with commercialized antibiotics should be carried out to determine more of its action. It was discussed that grifolin has a potential anticancer effect on different human cancer cell lines with numerous mechanisms, which open the challenge for the eventual use of this drug in chemotherapy and chemoprevention. However, despite its specific molecular actions, further pharmacokinetic studies investing in the toxicity, absorption, bioavailability, metabolism and elimination of grifolin are required to validate its safety and pharmacological response.

## Figures and Tables

**Table 1:** Sources of grifolin.

Plants/Mushrooms	Origin of Grifolin (Extracts/Essential Oils/Extracts of Mushrooms)	References
*Peperomia galioides*	Extract of plant	[7]
*Peperomia galioides*	Extract of plant	[19]
*Peperomia galioides*	Extract of plant	[8]
*Rhododendron dauricum*	Extract of plant	[20]
*Rhododendron dauricum*	Extract of plant	[9]
*Rhododendron dauricum*	Extract of plant	[21]
*Rhododendron dauricum*	Extract of plant	[22]
*Solanum lycopersicum*	Extract of plant	[23]
*Kayea assamica*	Extract of plant	[24]
*Albatrellus ovinus*	Extract of mushrooms	[25]
*Albatrellus ovinus*	Extract of mushrooms	[26]
*Albatrellus flettii*	Extract of mushrooms	[27]
*Albatrellus caeruleoporus*	Extract of mushrooms	[28]
*Albatrellus confluens*	Extract of mushrooms	[29]
*Albatrellus dispansus*	Extract of mushrooms	[30]

**Table 2:** Anticancer activity of Grifolin.

Cancer Type	Cell Lines	Experimental Approaches	Mechanism of Action	References
Marmoset B lymphoblastoid	B95-8	Flow cytometry Fluorescent staining	Apoptosis IC_50_ = 24 μM Decreased Bcl-2 expression	[30]
Burkitts lymphoma	Raji	Flow cytometry Fluorescent staining	Apoptosis IC_50_ = 27 μM Decreased Bcl-2 expression	
Chronic myelogenous leukemia	K562	Flow cytometry Fluorescent staining	Apoptosis IC_50_ = 18 μM Decreased Bcl-2 expression	
Colon cancer	SW480	Flow cytometry Fluorescent staining	Apoptosis IC_50_ = 27 μM Decreased Bcl-2 expression	
Nasopharyngeal carcinoma	CNE1	Flow cytometry Fluorescent staining	Apoptosis IC_50_ = 24 μM Released cytochrome c Decreased Bcl-2 expression	
Breast cancer	MCF7	Flow cytometry Fluorescent staining	Apoptosis IC_50_ = 30 μM Decreased Bcl-2 expression	
Cervical cancer	HeLa	Flow cytometry Fluorescent staining	Apoptosis IC_50_ = 34 μM Decreased Bcl-2 expression	
Murine macrophages	RAW 264.7	LPS-induced production of nitric oxide (NO)	Inhibited NO production IC_50_ = 29.0 μM	[31]
Nasopharyngeal carcinoma	CNE1	Flow cytometry Western blotting	Induced cell-cycle arrest in G_1_ phase via the ERK1/2 pathway	[34]
Osteosarcoma	MG63 and U2OS	Flow cytometry Western blotting	Apoptosis Inhibited PI3K/AKT signaling pathway	[32]
Lung cancer	A549	Colorimetric sulforhodamine B method	Cytotoxic effect 5.0 < IC_50_ < 10.5 μg/mL	[33]
Human melanoma	SK-Mel-2	Colorimetric sulforhodamine B method	Cytotoxic effect 8.0 < IC_50_ < 16.9 μg/mL	
Mouse melanoma	B16F1	Colorimetric sulforhodamine B method	Cytotoxic effect 3.5 < IC_50_ < 7.3 μg/mL	
Nasopharyngeal carcinoma	CNE1	Flow cytometry Western blotting	Upregulated DAPK1 via p53	[35]
Cervical cancer	HeLa	In vitro kinase assay Immunofluorescence analysis	Inhibited ERK1/2 kinase activities	[36]
Breast cancer	MCF7 and MDA-MB-231	In vitro kinase assay Immunofluorescence analysis	Inhibited ERK1/2 kinase activities	
Nasopharyngeal carcinoma	5-8F	In vitro kinase assay Immunofluorescence analysis	Downregulated the level of DNMT1 mRNA	
Nasopharyngeal carcinoma	5-8F	Metastatic mice (in vivo)	Reduced lung metastases to 18.2%	
Ovarian cancer	SKOV3 and A2780	Flow cytometry Western blotting	Apoptosis Inhibited the Akt/mTOR/S6K pathway	[37]
Nasopharyngeal carcinoma	5-8F	Cell adhesion assay Western blotting	Inhibited adhesion and migration of tumor cells	[38]
Gastric carcinoma	MGC-803	Cell adhesion assay Western blotting	Inhibited adhesion and migration of tumor cells	
Gastric cancer	SGC-7901 and BGC-823	Flow cytometry Western blotting Cell cycle assay	Apoptosis Inhibited the MAP kinase pathway	[39]
Ovarian cancer	A2780	Flow cytometry Western blotting	Apoptosis Decreased the expression of ERK1/2 and Akt	[41]
Gastric cancer	SGC-7901 and BGC-823	q-RT PCR assay Cell cycle arrest	Apoptosis Inhibited cell development and invasion	[40]
Gastric cancer	SGC-7901 and BGC-823	Xenografted nude BALB/c mice with gastric cancer cells	Increased the survival rate	
Nasopharyngeal carcinoma	CNE1 and C666-1	DNMT activity measurement	Inhibited the activity and expression of DNMT1	[14]
Pituitary adenoma	GH3	Flow cytometry Western blotting Cellular ATP measurement	Apoptosis IC_50_ = 4.25 μmol/L Inhibited the ATP production	[15]
Colon cancer	HT-29	Cytotoxicity assay Flow cytometry	Anti-cell viability effect IC_50_ = 35.4 ± 2.4 μM	[16]
Colon cancer	SW-480	Cytotoxicity assay Flow cytometry	Anti-cell viability effect IC_50_ = 27.4 ± 2.2 μM	
Cervical cancer	HeLa	Cytotoxicity assay Flow cytometry	Anti-cell viability effect IC_50_ = 30.7 ± 1.0 μM	

## Data Availability

Not applicable.
